# In Acute Stroke, Can CT Perfusion-Derived Cerebral Blood Volume Maps Substitute for Diffusion-Weighted Imaging in Identifying the Ischemic Core?

**DOI:** 10.1371/journal.pone.0133566

**Published:** 2015-07-20

**Authors:** William A. Copen, Livia T. Morais, Ona Wu, Lee H. Schwamm, Pamela W. Schaefer, R. Gilberto González, Albert J. Yoo

**Affiliations:** 1 Department of Radiology, Division of Neuroradiology, Massachusetts General Hospital, Boston, Massachusetts, United States of America; 2 Department of Neurology, Massachusetts General Hospital, Boston, Massachusetts, United States of America; 3 Harvard Medical School, Boston, Massachusetts, United States of America; University of Regensburg, GERMANY

## Abstract

**Background and Purpose:**

In the treatment of patients with suspected acute ischemic stroke, increasing evidence suggests the importance of measuring the volume of the irreversibly injured “ischemic core.” The gold standard method for doing this in the clinical setting is diffusion-weighted magnetic resonance imaging (DWI), but many authors suggest that maps of regional cerebral blood volume (CBV) derived from computed tomography perfusion imaging (CTP) can substitute for DWI. We sought to determine whether DWI and CTP-derived CBV maps are equivalent in measuring core volume.

**Methods:**

58 patients with suspected stroke underwent CTP and DWI within 6 hours of symptom onset. We measured low-CBV lesion volumes using three methods: “objective absolute,” i.e. the volume of tissue with CBV below each of six published absolute thresholds (0.9–2.5 mL/100 g), “objective relative,” whose six thresholds (51%-60%) were fractions of mean contralateral CBV, and “subjective,” in which two radiologists (R1, R2) outlined lesions subjectively. We assessed the sensitivity and specificity of each method, threshold, and radiologist in detecting infarction, and the degree to which each over- or underestimated the DWI core volume. Additionally, in the subset of 32 patients for whom follow-up CT or MRI was available, we measured the proportion of CBV- or DWI-defined core lesions that exceeded the follow-up infarct volume, and the maximum amount by which this occurred.

**Results:**

DWI was positive in 72% (42/58) of patients. CBV maps’ sensitivity/specificity in identifying DWI-positive patients were 100%/0% for both objective methods with all thresholds, 43%/94% for R1, and 83%/44% for R2. Mean core overestimation was 156–699 mL for objective absolute thresholds, and 127–200 mL for objective relative thresholds. For R1 and R2, respectively, mean±SD subjective overestimation were -11±26 mL and -11±23 mL, but subjective volumes differed from DWI volumes by up to 117 and 124 mL in individual patients. Inter-rater agreement regarding the presence of infarction on CBV maps was poor (kappa = 0.21). Core lesions defined by the six objective absolute CBV thresholds exceeded follow-up infarct volumes for 81%-100% of patients, by up to 430–1002 mL. Core estimates produced by objective relative thresholds exceeded follow-up volumes in 91% of patients, by up to 210-280 mL. Subjective lesions defined by R1 and R2 exceeded follow-up volumes in 18% and 26% of cases, by up to 71 and 15 mL, respectively. Only 1 of 23 DWI lesions (4%) exceeded final infarct volume, by 3 mL.

**Conclusion:**

CTP-derived CBV maps cannot reliably substitute for DWI in measuring core volume, or even establish which patients have DWI lesions.

## Introduction

Measurement of the ischemic core is among the most clinically important and widely pursued goals in acute stroke imaging. The core, which sometimes but not always is located near the center of an ischemic region, is defined as brain tissue that already has been irreversibly injured by ischemia at the time of imaging. Accumulating evidence supports selecting patients for arterial recanalization therapy based upon imaging-based measurement of the volume of the core. When the core is large, the potential improvement in a patient’s clinical outcome is limited,[1, 2] and recanalization therapies may actually worsen outcomes for patients with large cores,[3] perhaps because reperfusion of severely injured tissue aggravates vasogenic edema,[4] and/or increases the risk of bleeding from damaged blood vessels.[5]

Currently, the most widely accepted gold standard for delineation of the ischemic core in the clinical setting is diffusion-weighted magnetic resonance imaging (DWI). However, researchers have sought an imaging technique that can identify the core using computed x-ray tomography (CT) rather than magnetic resonance imaging (MRI), chiefly because CT scanners are more widely available than MRI scanners.[6]

In one frequently proposed CT-based approach, CT perfusion imaging is used to create maps of regional cerebral blood volume (CBV), and low-CBV lesions are presumed to be equivalent to those that would be seen on DWI.[7, 8] This approach has been adopted by at least one major multicenter trial of recanalization therapies,[9] and its validity is asserted by several organizations’ imaging recommendations and treatment guidelines.[10, 11] However, the reliability of CBV maps as a substitute for DWI is called into question by reports that CBV maps often fail to identify DWI lesions,[12–14] and that CBV is often elevated, rather than reduced, in DWI-abnormal tissue.[15–18] In this study, we tested the hypothesis that CT-derived CBV maps can be used reliably as a substitute for DWI in identifying and measuring the size of the ischemic core. To do so, we measured the sizes of 58 acute stroke patients’ low-CBV lesions using several alternative methods, and compared these volumes to those of the patients’ DWI lesions, in order to assess whether the CBV and DWI lesion volumes were equivalent. Additionally, in a subset of those patients for whom follow-up images were available, we assessed how often and to what the extent initial core volumes estimated by CBV and DWI must have been inaccurate, in that they exceeded follow-up infarct volumes.

## Materials and Methods

Review of patients’ images and medical records for this study was authorized by the Partners Human Research Committee, which is the Institutional Review Board of Partners HealthCare. The informed consent requirement was waived because only retrospective review was performed.

### Patient Selection and Imaging

We identified all patients at our institution who underwent CTP and DWI within 60 minutes of each other for workup of suspected acute stroke during an 18-month period. In that period, performance of CTP followed immediately by DWI was part of our institution’s standard imaging protocol for all patients with suspected acute stroke. We included only patients with ongoing symptoms, who were known to be at neurologic baseline less than six hours before the completion of both scans, and who did not receive thrombolytic therapy prior to imaging.

We determined whether or not each patient who was selected for inclusion for the study underwent a second imaging examination that could be used to calculate follow-up infarct volume. Stroke patients do not routinely undergo follow-up imaging at our institution, and these follow-up examinations were performed only if they had been ordered by the patients’ physicians. CT or MRI examinations of the brain performed between 48 hours and one year after the end of the first examination were considered acceptable follow-up studies, and the examination closest to 30 days later was selected when more than one was available. If both CT and MR follow-up studies had been performed, MR was chosen preferentially. Follow-up studies were excluded if they demonstrated more than petechial acute hemorrhage within an infarct, as this could inflate the measured lesion volume.

CTP was performed using a 64-slice CT scanner (Lightspeed, General Electric Medical Systems), with a 4 mm-thick detector array, using a “toggling table” technique, in which image acquisition alternated between two adjacent 4 mm slabs. Images were acquired at each slice location every three seconds, with a scan duration of 66 seconds. Tube voltage and current were 80 kV and 500 mA, respectively. Sixteen slices were acquired with a slice thickness of 5 mm. Image dimensions were 512 x 512 pixels. For most patients (n = 51), a 250 cm field of view was used. For the other patients, fields of view were between 208 and 225 cm. Either 40 mL (n = 11) or 45 mL (n = 47) of iopamidol with an iodine concentration of 370 mg/mL (Isovue-370, Bracco Diagnostics, Monroe Township, NJ) was injected via a peripheral intravenous catheter at 7 mL/s, followed by a saline flush.

DWI utilized a 1.5 T scanner (Signa, General Electric Medical Systems, Milwaukee, WI), and a spin-echo echo-planar pulse sequence that incorporated two 180-degree refocusing pulses, in order to minimize eddy current-related warping artifacts.[19] TR was 5000 ms, with minimum TE (84.3 to 98.6 ms). Slice thickness and spacing were 5 mm and 1 mm, respectively. Field of view was 22 cm, with a 128x128 reconstruction matrix, zero-filled in k-space to yield 256x256-pixel images. Enough slices were prescribed to cover the entire brain. Diffusion-weighted images were acquired with a diffusion-weighting (“b-value”) of 1000 s/mm2, in 25 different directions, and three additional volumes were acquired without diffusion weighting. The duration of the DWI sequence was 2 minutes and 35 seconds. Isotropic DWI images and apparent diffusion coefficient (ADC) maps were created from these data.

Patients’ clinical data and images were anonymized with two different sets of unrelated numeric codes used for CTP and DWI images, respectively. CTP source images were post-processed using commercially available software (CT Perfusion 4D, General Electric Healthcare). Maps of regional cerebral blood volume (CBV), cerebral blood flow (CBF), mean transit time (MTT), and time to fitted impulse response function peak (Tmax) were created. “Base” noncontrast images were also created, by averaging together the images that were acquired before the arrival of the contrast bolus. The manufacturer’s default settings were used, including 2D image coregistration and “smart smoothing.” The software’s automatic algorithms were utilized to locate regions of interest (ROIs) for computing arterial input (AIF) and venous output (VOF) functions. If AIF ROIs were automatically placed outside of an artery, they were repositioned (prior to creation of any post-processed perfusion maps) within a normal-appearing, superoinferiorly oriented segment of an anterior cerebral artery or supraclinoid internal artery, in order to avoid partial volume effects. VOF ROIs placed outside of a vein were repositioned within a superoinferiorly oriented segment of the superior sagittal sinus.

Computation of all perfusion maps (CBV, CBF, MTT, and Tmax) was based upon mathematical derivation of an impulse residue function (IRF) from each pixel’s time-density function, and the single global AIF, using an algorithm based upon singular value decomposition deconvolution.[20] Delay correction was implemented by model-based estimation of tracer arrival time in each pixel, t_0_. CBF was computed as the value of the IRF at t_0_. MTT was computed as the first moment of the IRF beginning at t_0_. CBV was computed as the product of CBF and MTT. Tmax was derived as (MTT ÷ 2) + t_0_. Absolute hemodynamic measurements were derived using the venous output function for normalization.[21]

### Image Analysis

A neuroradiologist with seven years of subspecialty experience (hereafter called “R1”) manually outlined regions that appeared to have abnormally low CBV, and volumes of the outlined regions were computed. In order to simulate conditions under which CTP images might be interpreted clinically, R1 was also provided with and reviewed the other post-processed CT images (i.e. base, CBF, MTT, Tmax), and received a brief summary of each patient’s neurologic symptoms (e.g. “left face, arm and leg weakness”). MR images were not available. This same subjective lesion measurement process was independently repeated by a second neuroradiologist (“R2”), who also had seven years of subspecialty experience.

In a separate session performed several months after subjective CBV lesion measurement, DWI lesions identifying restricted diffusion were outlined using a semi-quantitative thresholding-based technique. In this process, DWI images and ADC maps were available, but CT images were not.

Subsequently, because CTP encompassed only 16 slices but DWI covered the entire brain, and because CTP and DWI slices could not be prescribed at exactly the same angle, DWI ROIs were compared to the non-contrast CTP “base” images, and then edited, when necessary, so that the ROI(s) no longer contained any parts of the brain that were not included in the CTP images. Post-processed CTP maps other than the “base” images were not available during this process.

“Objective absolute” lesion volumes were calculated by measuring the number of voxels below an absolute CBV threshold, and multiplying the total by the voxel dimensions, using custom-written software. Background and CSF pixels were excluded. This analysis was repeated using six threshold values (0.9, 1.1, 1.3, 2.0, 2.2, and 2.5 mL/100 g) that have been reported by various earlier studies as best yielding CBV lesion volumes that approximate DWI lesion volumes [22–25].

Another custom-written computer program performed “objective relative” CBV lesion measurement. This program first measured the mean CBV in brain pixels in the contralateral side of the brain, i.e. the side opposite the DWI lesion, or opposite the majority of DWI lesions for patients with multiple lesions, or opposite the side on which a lesion would best explain the patient’s deficits if no DWI lesion was present. This program then measured the volume of all voxels on both sides of the brain for which CBV was lower than a threshold defined as a particular fraction of the mean contralateral CBV value. For each patient, this analysis was repeated using six different thresholds (51%, 55%, 56%, 58%, 59%, and 60% of the mean contralateral CBV value) that have been previously proposed as producing lesion volumes that best approximate DWI lesion volumes [22–25].

**Fig 1:**
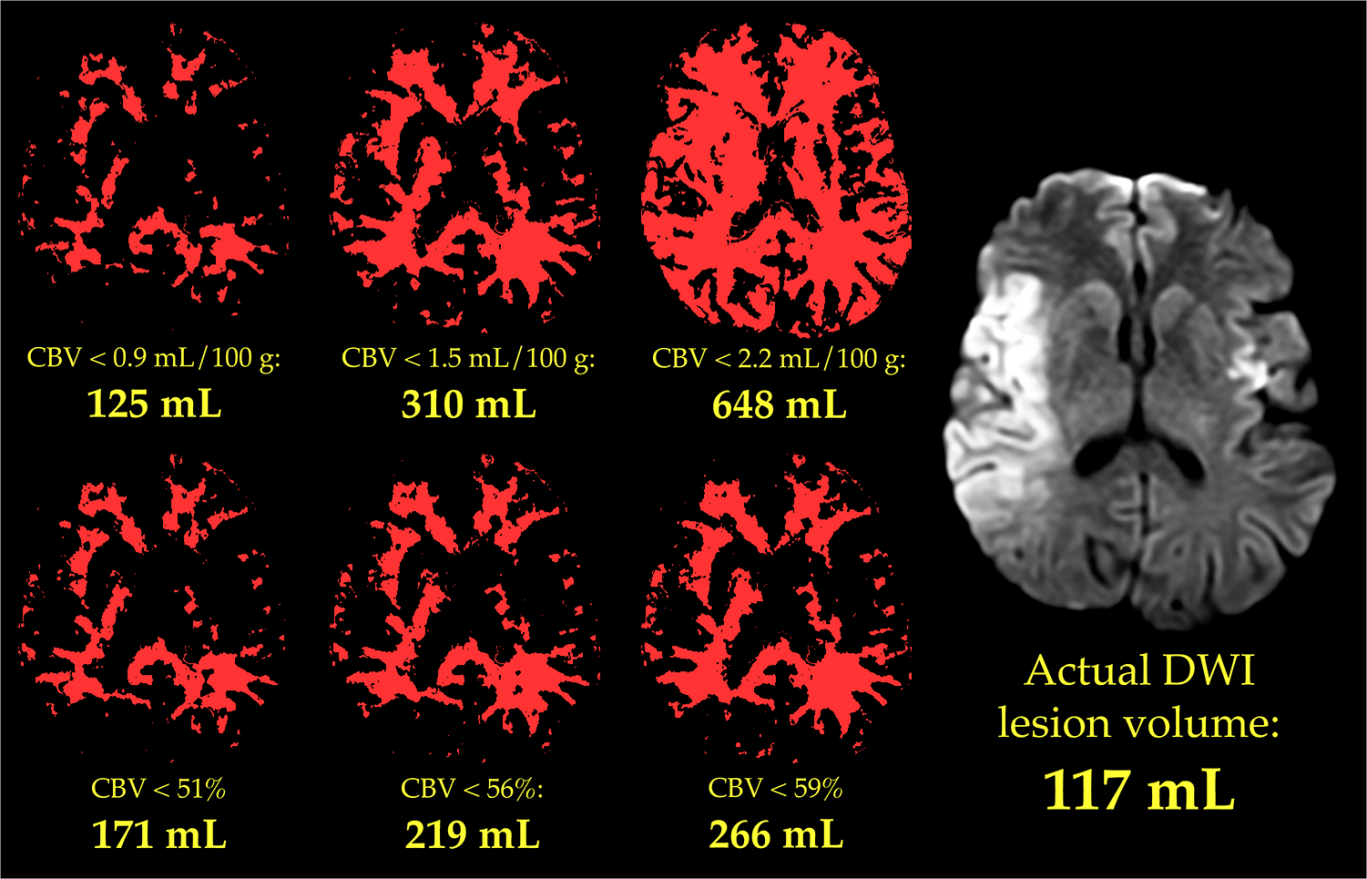
Objective measurement of CBV lesion volume. The six smaller images depict low-CBV lesions that were delineated automatically, in a single slice of one patient’s CBV maps, by rendering in white all brain pixels in which CBV was below each of six different thresholds. Each threshold is listed below its corresponding image, along with the total volume of tissue with CBV below that threshold in all slices. For clarity, the lesions identified by only three of the six absolute thresholds are shown in the top row, and lesions identified by only three of the six relative thresholds are shown in the bottom row. The larger image on the right is a DWI image obtained from the same patient, at approximately the same level. The gold standard core volume, measured over all DWI slices, was 117 mL.

**Fig 2:**
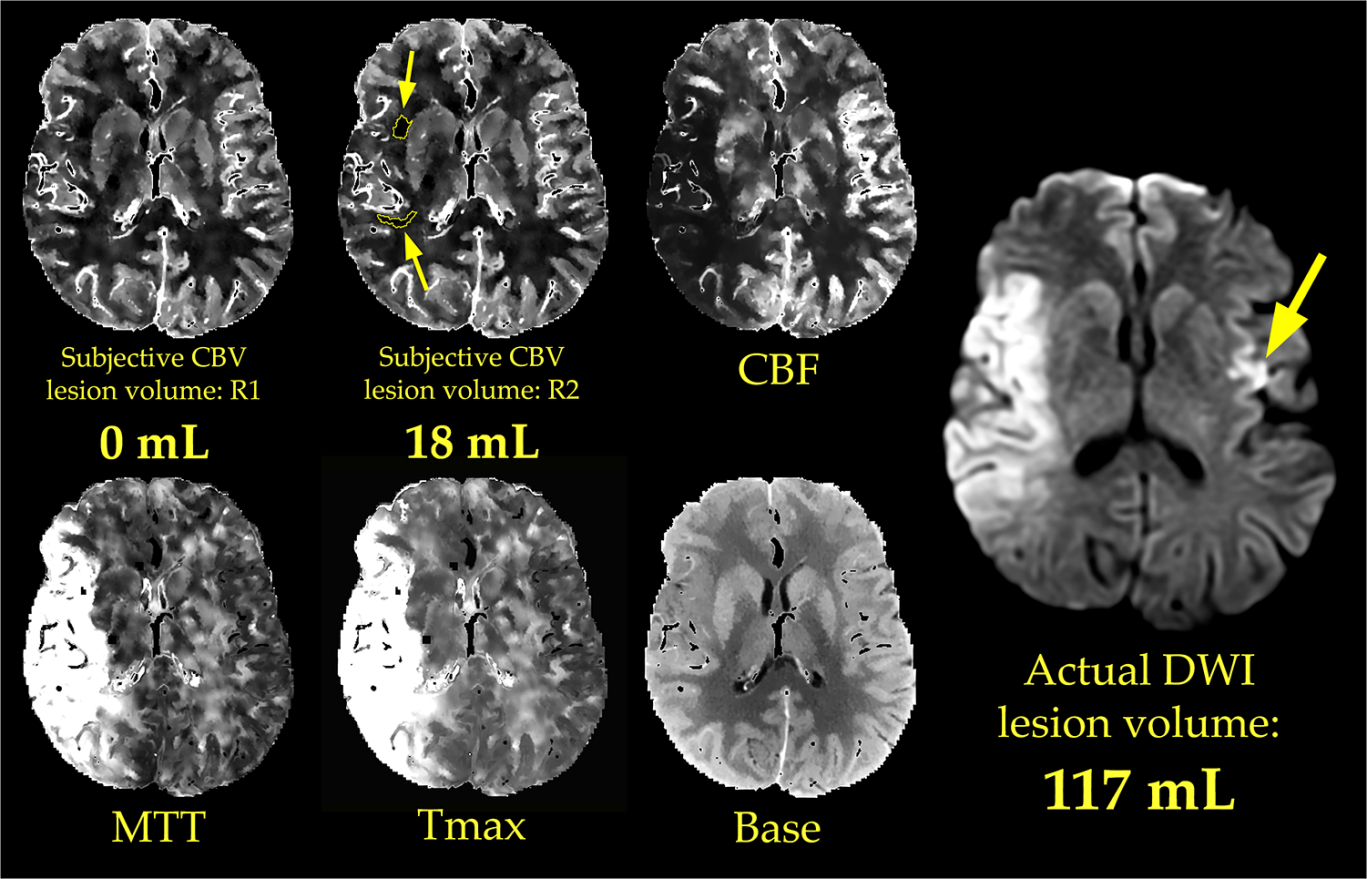
Subjective measurement of CBV lesion volume. Two radiologists (R1 and R2) subjectively outlined regions of abnormally low CBV. In doing so, the radiologists had access to all available CTP images, and to clinical history (in this case, “left face, arm, and leg weakness and left hemianopia”). The patient and slice location are the same as those in Fig 1. R1 did not detect a lesion, i.e. he measured the core lesion volume to be 0 mL. R2 outlined several lesions (yellow outlines and small arrows) with a total volume of 18 mL. R2 failed to identify the presence of infarction in the left cerebral hemisphere (large yellow arrow in the DWI image), which suggested an embolus of central origin rather than one arising from the right internal carotid artery.

**Fig 3:**
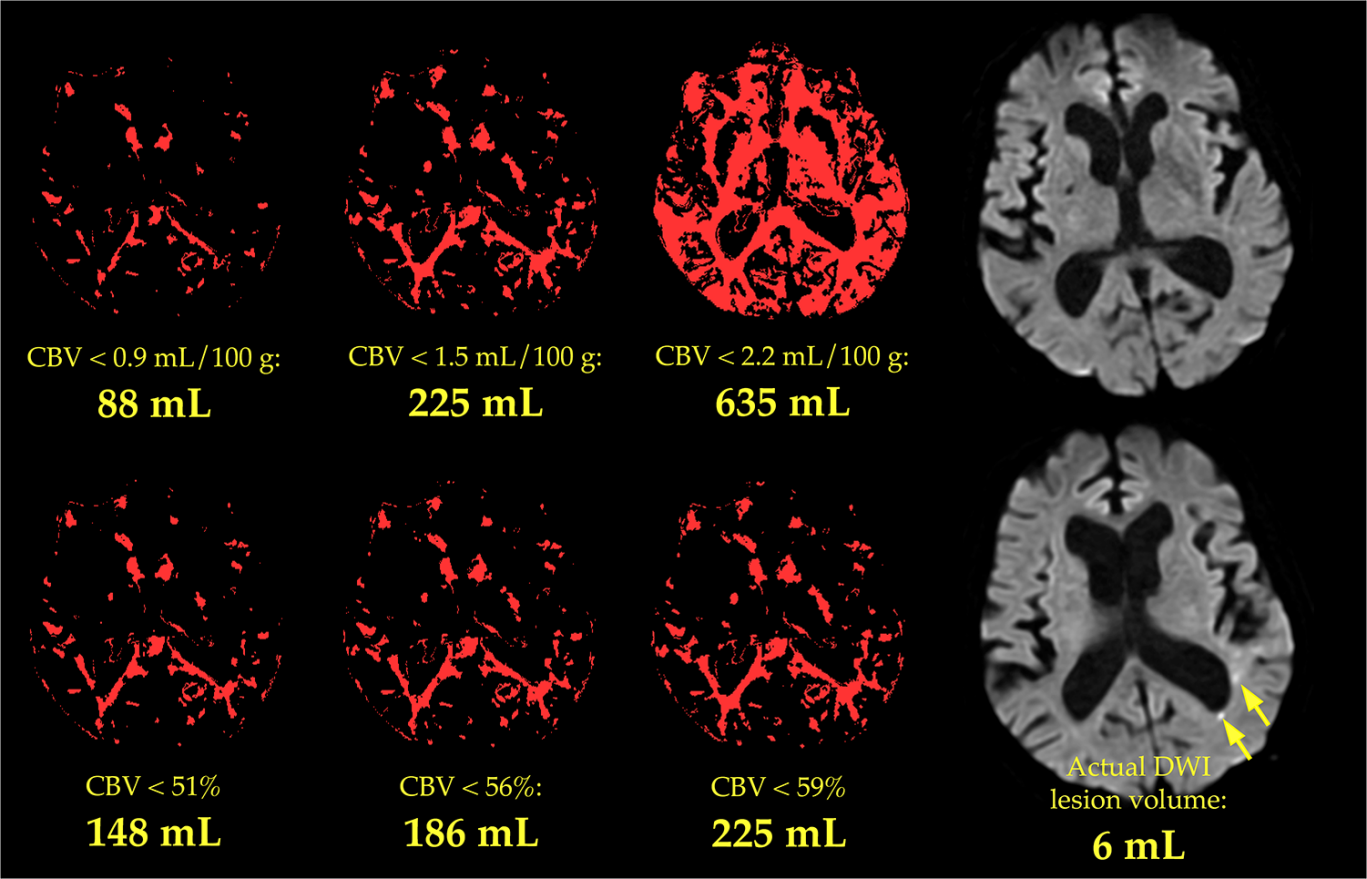
Objective measurement of CBV lesion volume in a second patient. The six smaller images depict low-CBV lesions that were delineated automatically, in a single slice of a second patient’s CBV maps, by rendering in white all brain pixels in which CBV was below each of six different thresholds. Each threshold is listed below its corresponding image, along with the total volume of tissue with CBV below that threshold in all slices. For clarity, the lesions identified by only three of the six absolute thresholds are shown in the top row, and lesions identified by only three of the six relative thresholds are shown in the bottom row. No lesions were seen in a DWI image acquired at approximately the same level (upper right), although DWI images acquired more superiorly showed several small lesions whose total volume was 6 mL (bottom right, arrows).

**Fig 4:**
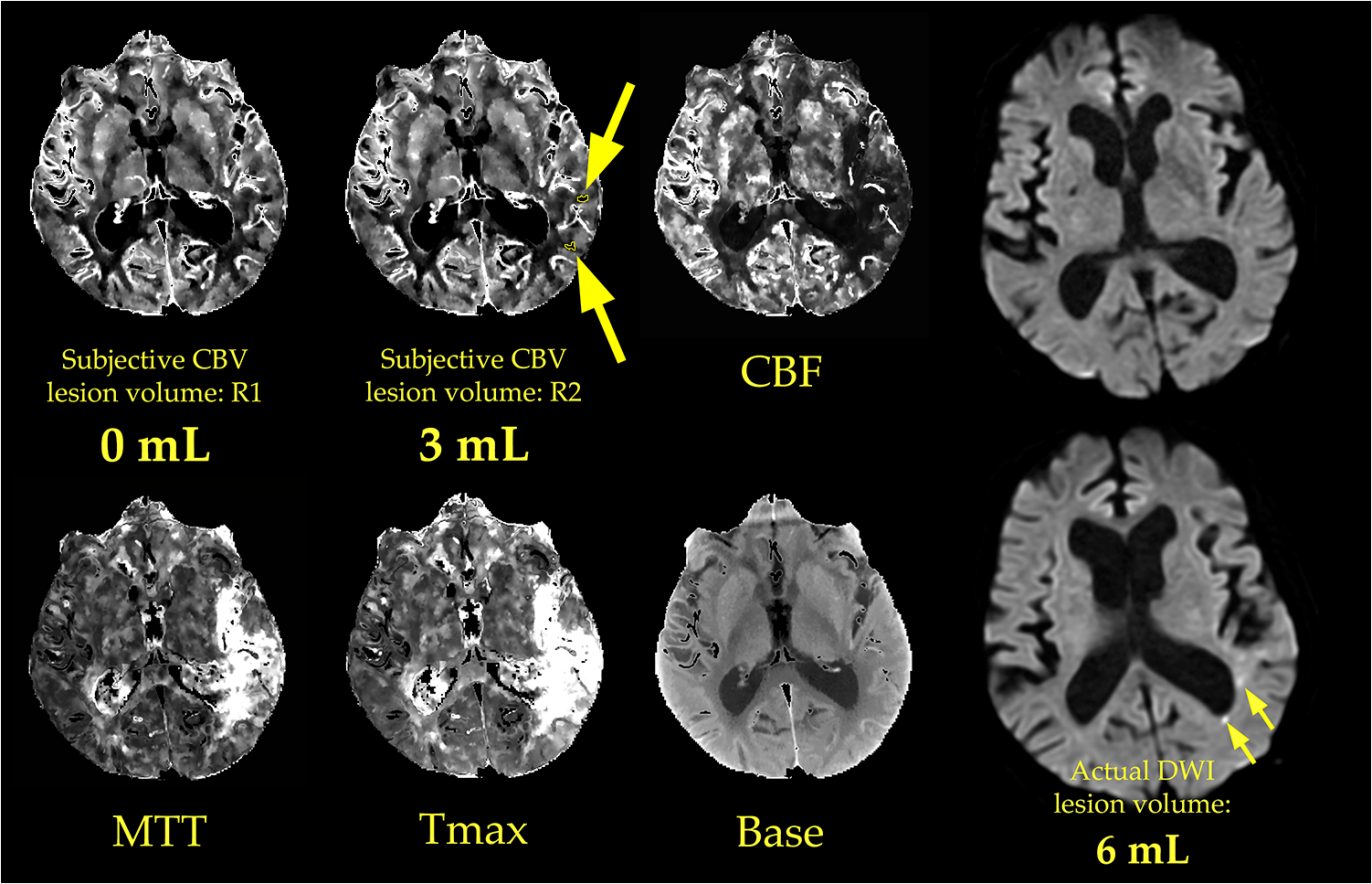
Subjective measurement of CBV lesion volume in a second patient. Two radiologists (R1 and R2) subjectively outlined regions of abnormally low CBV. In doing so, the radiologists had access to all available CTP images, and to clinical history (in this case, “aphasia and right face, arm, and leg weakness”). The patient and CT slice location are the same as those in Fig 3. R1 did not detect a lesion, i.e. he measured the core lesion volume to be 0 mL. R2 outlined several lesions (yellow outlines and large arrows) whose total volume was 3 mL. Although R2’s subjective lesion volume was very close to the actual DWI lesion volume of 6 mL, none of the CBV lesions that R2 outlined corresponded to actual DWI lesions. No lesions were present in a DWI image obtained at approximately the same level as these CTP maps (upper right), and all of the DWI lesions that were present were located more superiorly (lower right, small arrows).

Examples of objective and subjective lesion assessment in one patient are provided in Figs 1 and 2, respectively. Examples from a second patient are presented in Figs 3 and 4, respectively.

Follow-up lesions were outlined using the same semi-automated process that was used to outline acute DWI lesions. For CT follow-up studies, lesions were measured in noncontrast images. For MR follow-up studies, the larger of the lesions seen in DWI images and T2-weighted fluid-attenuated inversion recovery (FLAIR) images was measured. For all follow-up studies, measured lesion volumes included only those portions of the lesion(s) lying within the part of the brain that had been included in the initial CTP acquisition.

### Statistical Analysis

For each of the three CBV lesion identification methods (objective absolute, objective relative, and subjective), and for each of the objective threshold levels used for the objective methods, and each of the two radiologists who measured lesions subjectively, the sensitivity and specificity of CBV maps for identifying patients with DWI-positive infarction was computed. True positives were defined as patients in which both CBV and DWI lesions (irrespective of their volumes) were identified. False positive patients had CBV lesion volumes greater than zero, but no DWI lesion. True negative patients were defined as those whose CBV maps and DWI images showed no lesions. False negative patients were those whose CBV maps showed no lesions, but lesions were evident on DWI.

For each method and threshold, the correlation between CBV and DWI lesion volumes was computed using the Pearson product-moment correlation coefficient, and the statistical significance of the correlation was tested using a two-tailed p-value threshold of 0.05. “CBV overestimation volume” was computed for each combination of patient, method, and threshold (for objective methods) or radiologist (for the subjective method), by subtracting the true DWI lesion volume from the CBV lesion volume. The mean, median, minimum, and maximum of all patients’ overestimation volumes were computed. Inter-rater agreement between R1 and R2 with respect to the presence versus absence of a CBV lesion was assessed using Cohen’s kappa statistic.

The subsets of patients who did and did not undergo follow-up examinations were compared to each other with respect to age, sex, National Institutes of Health Stroke Scale (NIHSS) score, initial DWI lesion size, and likelihood of having a DWI lesion, in order to assess whether the patients with follow-up studies constituted a representative subset of the larger patient sample. The groups’ ages, NIHSS scores, and initial DWI lesion sizes were compared with two-tailed t-tests. Sex and likelihood of having a DWI lesion were compared using Pearson’s chi-square test.

Any initial core volume estimate that is smaller than the follow-up infarct volume may or may not have been an accurate indicator of the true ischemic core, even if the follow-up lesion is very much larger. However, by definition, an initial core estimate is inaccurate if it proves to be larger than the follow-up infarct volume. Therefore, in order to provide an indirect assessment of the various methods’ validity in measuring the true core, we computed the frequency with which the volumes of initially detected core lesions exceeded the follow-up infarct volumes, and the maximum amount by which this occurred, for each CBV method, threshold, and radiologist, and for DWI.

## Results

Of 66 patients who satisfied the inclusion criteria, eight were excluded because their DWI (n = 2) or CTP (n = 4) images were too motion-degraded to be interpretable, or because of a CT scanner malfunction (n = 1) or technologist error (n = 1). Images from the remaining 58 patients were analyzed. They ranged in age from 38.2 to 94.2 years (mean 69.2), and 35 were male. NIHSS scores were recorded for 56 of these patients, and the median NIHSS score was 8, with an interquartile range of 2.5–16.5. CTP was performed between 0.48 and 5.80 (mean 3.43) hours after the time when the patient was last seen at neurologic baseline, and DWI was performed between 0.95 and 5.92 (mean 3.78) hours of that time. CTP preceded DWI for all patients, and the mean and median times between CTP and DWI were 19.5 and 16.5 minutes, respectively.

Of the 58 patients included in the study, 33 had follow-up imaging examinations. The images from one patient were excluded because of hemorrhage, so that follow-up lesion volumes were calculated for 55% (32/58) of patients. One patient from each of the two subsets did not have a recorded NIHSS score. Attributes of the subsets of patients who did and did not have follow-up studies are compared in Table 1. No statistically significant group-related differences were found between patients who did and did not undergo follow-up imaging.

**Table 1:** Comparison of the subsets of patients who did and did not have follow-up examinations.

	Follow-up	No follow-up	Difference
n	32	26	n/a
Mean age	69.2	73.6	*p* = 0.97
Sex	59% (19/32) male	62% (16/26) male	*p* = 0.87
Mean/median NIHSS (interquartile range)	9.9/8 (2–23)	10.1/8 (3–23)	*p* = 0.91
DWI positive	72% (23/32)	73% (19/26)	*p* = 0.92
Mean/median DWI lesion size (mL)	21.9/5.2	33.3/5.8	*p* = 0.49

DWI scans were positive in 72% (42/58) of patients. DWI lesions extended outside of CTP volumes, necessitating truncation of DWI lesion ROIs, for 29% (17/58) of patients. This adjustment reduced DWI lesion volumes by less than 1 mL for 10 of the 17 patients, and less than 4 mL for 13 patients. The mean and median of adjustment volumes were 6.8 and 0.8 mL, respectively. Following adjustment, the mean and standard deviation of all patients’ DWI lesion volumes (including those for whom lesion volume was zero) were 37.3 and 70.9 mL, respectively.

Table 2 lists the sensitivities and specificities of the various CBV lesion assessment methods for detecting patients with DWI-positive infarcts, as well as correlations between measured CBV lesion volume and “gold standard” DWI lesion volumes for each method, and the mean, median, and range of overestimation volumes for each method. All of the objective threshold-based CBV lesion measurement methods were 100% sensitive and 0% specific in identifying patients with DWI lesions, in that, regardless of which threshold was used, and whether or not there was any actual DWI lesion, there were always some pixels with CBV values below the thresholds investigated in this study.

**Table 2:** Comparison of core lesion measurement methods.

Method	Threshold	Sensitivity^1^	Specificity^1^	Correlation between CBV and DWI lesion volumes (p-value)	Mean/Median DWI lesion overestimation, mL	Range of DWI lesion overestimation, mL	Core lesion exceeding follow-up: frequency	Core lesion exceeding follow-up: maximum (mL)
Absolute	0.9 mL/100 g	100%	0%	0.07 (p = 0.58)	156/136	-79 to 919	81% (26/32)	430
Absolute	1.1 mL/100 g	100%	0%	0.01 (p = 0.94)	242/224	-55 to 943	94% (30/32)	614
Absolute	1.3 mL/100 g	100%	0%	-0.05 (p = 0.71)	331/318	-36 to 959	94% (30/32)	762
Absolute	2.0 mL/100 g	100%	0%	-0.17 (p = 0.18)	586/628	67 to 985	94% (30/32)	970
Absolute	2.2 mL/100 g	100%	0%	-0.19 (p = 0.16)	638/677	104 to 1001	94% (30/32)	986
Absolute	2.5 mL/100 g	100%	0%	-0.20 (p = 0.14)	699/747	159 to 1043	100% (32/32)	1002
Relative	51% of contralateral	100%	0%	0.36 (p = 0.006)	127/129	-0.1 to 608	91% (29/32)	210
Relative	55% of contralateral	100%	0%	0.35 (p = 0.007)	159/156	15 to 608	91% (29/32)	254
Relative	56% of contralateral	100%	0%	0.37 (p = 0.004)	166/158	31 to 608	91% (29/32)	254
Relative	58% of contralateral	100%	0%	0.38 (p = 0.004)	180/174	31 to 608	91% (29/32)	280
Relative	59% of contralateral	100%	0%	0.39 (p = 0.003)	188/191	49 to 608	91% (29/32)	280
Relative	60% of contralateral	100%	0%	0.35 (p = 0.008)	200/200	49 to 608	91% (29/32)	280
Subjective, R1	n/a	43%	94%	0.91 (p < 0.001)	-11/-5	-117 to 115	18% (2/11)	71
Subjective, R2	n/a	83%	44%	0.93 (p < 0.001)	-11/-2	-124 to 15	26% (7/27)	15
DWI	n/a	n/a	n/a	n/a	n/a	n/a	4% (1/23)	3

Notes: (1) Sensitivity and specificity refer to CBV maps’ ability to identify patients with true DWI lesions, irrespective of lesion volumes. (2) “Core overestimation” refers to the quantity by which CBV lesion volume exceeded true DWI lesion volume.

None of the six objective absolute CBV thresholds yielded a statistically significant correlation between CBV and DWI lesion volumes. Objective absolute lesion size measurements overestimated DWI lesion volume by an average of between 156 mL and 699 mL, depending upon the threshold used.

The objective relative method produced weak but significant correlations (*r* = 0.35–0.39) between CBV and DWI lesion volumes for all six thresholds tested, and overestimated infarct volume by an average of between 127 and 200 mL, depending upon the threshold used.

The subjective assessment method yielded lesion volume correlation coefficients that were statistically significant, and higher than those of any of the objective methods: 0.91 and 0.93, for R1 and R2, respectively. However, the radiologists’ subjective lesion volume assessments differed from DWI lesion volumes by as much as 115 mL for R1, and as much as 124 mL for R2. In subjectively assessing CBV maps, R1 was 43% sensitive and 94% specific in detecting patients who had DWI-positive infarction, whereas R2 was 83% sensitive and 44% specific. The two radiologists agreed with one another regarding whether or not a lesion was present in CBV maps for 53% of patients (31/58), with a kappa statistic of 0.21, indicating poor inter-rater reliability.

The next-to-rightmost column in Table 2 lists the proportion of initial core lesions detected by all CBV methods, and by DWI, that exceeded the follow-up infarct volume. Note that the denominator in each proportion is the number of patients with follow-up imaging for whom core lesions were detected, not the total number of patients. The results are presented in this manner in order to provide a more meaningful description of core estimation accuracy. For example, a method that never detected any core lesion at all would never exceed the follow-up infarct volume, but it would be misleading to imply that such a method was an accurate one.

The rightmost column in Table 2 lists the maximum volume by which each method’s initial core estimate exceeded final infarct volume.

The core lesion volumes produced by the objective absolute CBV method exceeded follow-up infarct volumes in 81%-100% of patients, and by up to 430–1002 mL, depending upon the threshold that was used. Volumes produced by the objective relative method exceeded follow-up infarct volumes in 91% of patients, and by up to 210–280 mL, depending upon the threshold. The subjective CBV lesions measured by R1 and R2 exceeded follow-up volumes in 18% and 26% of cases, and by up to 71 mL and 15 mL, respectively. The gold standard method for core estimation, DWI, produced 23 lesions, of which one (4%) exceeded the final infarct volume, by 3 mL.

## Discussion

This study’s principal finding was that, in the imaging evaluation of acute stroke patients, CTP-derived maps of CBV cannot reliably substitute for DWI in measuring the size of the ischemic core, or even determine whether or not a patient has a DWI lesion. Objective thresholding of CBV maps using a variety of different published thresholds yielded core lesion volume measurements that greatly exceeded actual DWI lesion volumes, and, when absolute thresholds were used, no significant correlation between CBV and DWI lesion volumes was detected. Much higher correlation coefficients were found when two experienced neuroradiologists outlined low-CBV lesions subjectively: 0.91 and 0.93, respectively. These coefficients are similar to those reported by previous studies that are widely cited as justification for the use of CBV maps in place of DWI.[7, 8] However, correlation between two measurement methods does not imply their interchangeability,[26] and our results highlight the potential danger in this fallacy. Despite our replicating previously reported correlations between subjective CBV volumes and true DWI lesion volumes, these measurements differed by over 100 mL in individual patients, and the two radiologists agreed in only 53% of cases regarding whether an infarct was present at all.

Our secondary finding was that CBV-derived core volume estimates were often inaccurate, in that they frequently exceeded follow-up infarct volumes, sometimes by hundreds of mL. In contrast, only one of 23 DWI lesions exceeded follow-up infarct volume, and by only 3 mL.

The discordance between lesions identified by DWI and CBV maps, and the frequent inaccuracy of CBV in core volume measurement, mirror fundamental disparities in the physiologic phenomena that these two imaging techniques depict. DWI identifies cytotoxic edema, a condition that results from the cumulative effects of sufficiently severe and prolonged hypoperfusion. When intracellular energy stores have been sufficiently depleted, energy-dependent membrane ion pumps fail, causing osmolytes and water to shift from the extracellular space to the intracellular space. This shift entails restrictions upon water molecules’ self-diffusion, and produces a visible DWI lesion.[27]

The onset of cytotoxic edema is a complex event that depends upon a variety of factors, including varying vulnerability to ischemic injury in different parts of the brain, ischemic preconditioning in patients with longstanding cerebrovascular disease, as well as dynamic fluctuations in regional perfusion pressure that have occurred acutely in the minutes and hours prior to a patient’s presentation, as systemic blood pressure fluctuated, and arterial emboli formed, migrated, and disintegrated. These factors cannot be evaluated by any imaging technique. However, once their downstream effects have caused cytotoxic edema and therefore a DWI lesion, cellular survival is unlikely. Although permanent reversal of DWI-abnormal tissue has been observed, this is an uncommon occurrence, and one that usually involves only a small volume of affected tissue.[28, 29] Therefore, DWI provides generally reliable, though imperfect identification of the ischemic core.

In comparison, CBV is a hemodynamic measurement, not a metabolic one. CBV is defined as the volume occupied by blood vessels within a particular part of the brain. Numerous experimental studies have observed that, in response to a drop in regional cerebral perfusion pressure (CPP), blood vessels may respond by quickly dilating. This response can help to preserve tissue viability by reducing cerebrovascular resistance and maintaining normal blood flow, or at least by increasing vascular transit time, thereby enabling increased tissue oxygen extraction.[30, 31] When autoregulatory vasodilation occurs, it may be detectable by imaging studies as an increase in CBV.

The use of CBV in identifying the ischemic core is based upon the premise that CBV falls to below-normal levels in irreversibly injured brain tissue, but not in other tissue. Some studies have indeed measured low CBV in severely ischemic tissue. However, the circumstances under which CBV falls below normal levels are poorly defined, and experimental data regarding CBV in the setting of very low perfusion pressure are lacking.[30] Most human perfusion imaging studies that have reported low CBV values have been performed with bolus-tracking techniques (e.g. CTP or MR perfusion imaging) that underestimate CBV in regions of low blood flow, to an extent that depends upon the duration of the scan and the post-processing algorithm that is used. It is possible that the phenomenon of apparently reduced CBV in ischemic tissue may be largely an artifact of the techniques that are used for hemodynamic measurement. [18, 31, 32]

Even when CBV truly falls, there is no physiologic rationale for the assertion that this occurrence should coincide with the moment when ischemic injury becomes irreversible. One of the most important discoveries of laboratory research on cerebral ischemia was the observation that the reversibility of ischemic injury is time-dependent, i.e. brain tissue progresses from “not core” to “core” from one moment to the next, without any hemodynamic change taking place.[30, 33, 34] This principle is the basis for much of modern acute stroke therapy, including the fundamental notion that brain tissue can be saved by timely arterial recanalization therapy. It implies that no hemodynamic measurement can identify the core, and therefore calls into question not only the use of CBV in that role, but also identification of the core using cerebral blood flow (CBF), which has been proposed in several recent studies.[23–25, 35, 36] In light of this logical inconsistency, we are assessing the equivalence of DWI lesions and CBF lesions in ongoing research.

Our study was performed at a single hospital, and included a relatively small number of patients. The generalizability of our findings would be enhanced by replication of our results in larger studies performed at different institutions. Also, we analyzed CBV maps that were produced by only a single post-processing program. Previous studies have found that results may vary substantially when CTP data are processed using different software, although the software-related differences reported for CBV calculations are generally smaller than those reported for other hemodynamic parameters. [20, 25, 37, 38] Replication of our results by future studies employing different software would be valuable, although the magnitude of the discrepancies between CBV and DWI lesions in the current study, and the absence of a proposed physiologic basis for the equivalence of CBV and water diffusion, suggest that similar results might be achieved.

Like any study that assesses the equivalence of CTP and DWI, ours is limited by the physical impossibility of performing both scans at exactly the same time. It is possible that physiologic changes occurring between the scans could change the size of the core, resulting in a discrepancy between the results of techniques that are actually equivalent. We attempted to minimize the likelihood of this by including only patients whose CTP and DWI scans were completed within one hour of each other. Future studies might attempt to address this problem by randomizing the order of CTP and DWI, or perhaps by repeating the first imaging study after completion of the second. However, in our study, as in all four previous studies from which our objective CBV thresholds were drawn,[22–25] CTP was always performed before DWI. Our study’s median CT-to-DWI time of 16.5 minutes was shorter than those of the aforementioned prior studies, which ranged from 25 to 34 minutes, suggesting that the likelihood of significant physiologic changes occurring between CTP and DWI would be no larger in our sample than in theirs.

The current study differs from most previously published research comparing CBV and DWI, in that we used the “toggling table” CTP technique, which doubles brain coverage at the expense of temporal resolution. Our use of the toggling table technique with three-second temporal resolution probably reflects widespread clinical practice, as this constitutes a typical, manufacturer-recommended clinical use of widely used CT scanners supplied by at least two major manufacturers, and one that is consistent with the detailed protocol recommendations of the American Association of Physicists in Medicine,[39] as well as current practice guidelines jointly issued by the American Society for Neuroradiology, American College of Radiology, and Society for Pediatric Radiology.[40] However, as this technique differs from that used in some prior studies, the effects of temporal resolution upon CTP-derived perfusion maps deserve consideration.

In a detailed analysis of those effects, Wintermark and colleagues found that longer sampling intervals such as ours tend to result in overestimation of regional CBV.[41] This suggests that, if we had used a shorter sampling interval, our CBV maps might have overestimated DWI lesion volumes even more than they did, at least when absolute CBV thresholds were tested. On the other hand, Wintermark et al. also found that, for a sampling interval of 3 seconds, significant overestimation of CBV could be avoided by injecting at least 40 mL of the contrast agent, which in their study had an iodine concentration of 300 mg/mL. In comparison, the scans in our study used 40 or 45 mL of contrast, with a higher iodine concentration of 370 mg/mL. This suggests that our findings would not have been influenced greatly by our relatively long sampling interval. Nevertheless, the generalizability of our findings would be strengthened by additional research replicating our results not only with different post-processing software, but also with different image acquisition techniques.

Like previous research comparing CBV and DWI lesion volumes, this study is limited by incomplete coverage of the brain in CTP images. Following the approach used by previous studies, [7, 8] we addressed this issue by excluding from DWI lesion volume measurements any brain tissue that was not included in CTP images. However, recent improvements in multidetector CT technology now enable complete or nearly complete CTP brain coverage with some scanners, potentially eliminating the possibility of CBV lesion volumes’ differing from DWI volumes solely because of differences in brain coverage. In our study, DWI lesions extended outside of CTP coverage by more than 4 mL in only 7% of patients. This suggests that complete brain coverage would not greatly alter our major findings.

In this study, subjective CBV lesion volumes were measured by two experienced radiologists, R1 and R2, who attempted to identify lesions as they would in routine clinical practice. However, because R1 and R2 were aware of the study’s hypothesis when making these measurements, it is possible that their estimates were unconsciously biased toward over- or underestimation. We limited the influence of any such bias as best we could, by forcing R1 and R2 to measure subjective lesion volumes without access to the DWI images. Nevertheless, as is the case for subjective assessment of lesion volumes in any research study, or in clinical practice, the possibility of unconscious bias cannot be entirely excluded.

Our data demonstrate that CBV maps cannot reliably substitute for DWI in measuring core volume, in that the two techniques may produce greatly different results. This finding alone does not address the question of which technique provides the more physiologically valid measurement. Our study’s secondary purpose was to address this question by assessing how often initial CBV and DWI lesions exceed follow-up infarct volumes, and therefore must have been physiologically inaccurate.

We found that CBV lesions were less accurate, in that they much more commonly exceeded follow-up infarct volumes, compared to DWI, and by much larger maximum amounts. However, this secondary finding must be interpreted with caution. Whereas performance of CTP and DWI were part of our institution’s routine imaging protocol for acute stroke patients, follow-up studies were performed only when deemed necessary by the patients’ physicians. Although we did not detect any significant differences between the patients who did and did not undergo follow-up imaging in our study, it is nevertheless possible that our patients with follow-up are not a representative sample of patients who present with acute stroke symptoms.

Also, our methodology is capable of detecting physiologic inaccuracy in core volume estimates only to the extent that those estimates exceed follow-up infarct volumes. Our study could not detect, for example, underestimation of core volume at the time of acute imaging. To do so would require confirming reperfusion of all ischemic brain tissue immediately after the initial imaging studies, and our study did not attempt to do this.

Our finding that CBV maps cannot substitute for DWI in identifying the ischemic core suggests that acute stroke research might most fruitfully focus on other clinical roles for perfusion imaging. Ischemic stroke is, by definition, primarily a disease of impaired perfusion, and accordingly, the wealth of hemodynamic information provided by perfusion imaging might serve a variety of worthwhile clinical purposes. These might include, for example, confirming that neurologic deficits of uncertain etiology have a vascular cause, determining whether there is or is not any region where there is a persistent reduction in perfusion pressure that might be remedied by arterial recanalization, guiding systemic blood pressure management or other “collateral therapies,” or distinguishing between reperfused and non-reperfused infarcts in order to inform treatment decisions regarding anticoagulation or mitigation of vasogenic edema. However, our results show that CBV maps derived from CT perfusion imaging cannot substitute for DWI in identification of the ischemic core.
